# Imaging features of double aortic arch shown by multidetector computed tomography angiography

**DOI:** 10.4103/0974-2069.74049

**Published:** 2010

**Authors:** Hetal N Jeeyani, Vaishali J Prajapati, Nehal H Patel, Shaunak B Shah

**Affiliations:** Department of Pediatrics, U N Mehta Institute of Cardiology and Research Centre, Ahmedabad, India; 1 Department of Cardiovascular and Thoracic Surgery, U N Mehta Institute of Cardiology and Research Centre, Ahmedabad, India

**Keywords:** Double aortic arch, multidetector computed tomography, stridor, vascular ring

## Abstract

We present a three-dimensional reconstructed image of vascular ring in a 2.5-month-old patient, which was obtained using multidetector computed tomography (MDCT). MDCT angiography made an accurate diagnosis of this life-threatening, but correctable, anomaly in an infant with a stridor, repeated respiratory infections and episodes of apnea.

## INTRODUCTION

Congenital anomalies are common causes of morbidity and mortality in pediatric population. Although double aortic arch is a rare congenital anomaly, it is an important cause of persistent respiratory symptoms and feeding difficulties in infants and children. Sometimes it can even lead to life threatening apneas. Hence, its early diagnosis and surgical management are necessary. Multidetector computerized tomography with 3 dimensional reconstruction has proved to be an important diagnostic modality which completely delineates the anatomy of double aortic arch and aids in surgical management.

## CASE REPORT

**Figure 1 (a,b):**
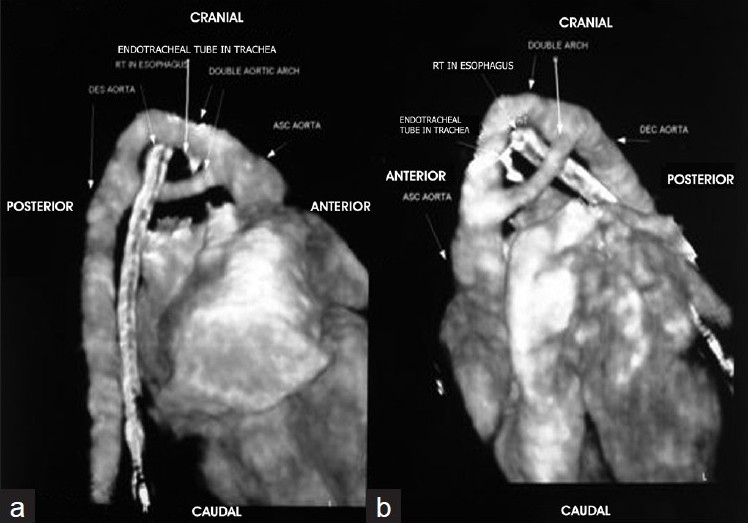
Multidetector computed tomography with 3-dimensional reconstruction images showing double aortic arch encircling the esophagus and trachea with the Ryle’ tube and endotracheal tube *in situ*, respectively

A 2.5-month-old male infant presented on ventilatory support with history of stridor since the neonatal period and episodes of apnea. The patient was a full-term baby with a birth weight of 3 kg, born by normal vaginal delivery. He was hospitalized at 1.5 months and 2 months of age with respiratory tract infections. During the second admission, he had two episodes of apnea for which he required ventilatory support. Chest X-ray showed bilateral hyperinflation with patchy consolidation. It did not show any evidence of common causes of stridor, like lobar emphysema, bronchogenic cyst or mediastinal mass. Because most common causes for stridor in the neonatal period include laryngeal and tracheal diseases, e.g. laryngomalacia, tracheomalacia, laryngeal and tracheal cysts, webs, stenosis and mucus plugs fibreoptic laryngoscopy and bronchoscopy were carried out. Bronchoscopy revealed external pulsatile compression on trachea. Hence, suspecting vascular ring, imaging was performed using 64-slice MDCT to confirm the diagnosis and determine further details. MDCT with three-dimensional (3D) reconstruction showed double aortic arch forming a vascular ring, encircling and compressing the trachea and esophagus [Figure 1a and b]. The right aortic arch was the dominant arch with right subclavian and right common carotid arteries arising from the right arch and left common carotid and left subclavian arteries arising from the left arch. Echocardiography did not show any evidence of double aortic arch because of suboptimum suprasternal acoustic window. However, it helped to rule out any associated intracardiac anomaly.

The infant underwent an emergency surgery through left posterolateral thoracotomy. Before the anomalous arch was removed, pulling endotracheal tube proximally from the site of constriction led to increase in airway pressure, which suggested that the anomalous arch led to constriction of trachea and the resultant symptoms. During surgery, the left aortic arch after the origin of the left common carotid and left subclavian arteries was removed and both ends were oversewn. Additionally, the ligamentum arteriosum was also divided. After removal of the anomalous left arch, the airway pressure did not rise on pulling the endotracheal tube proximally. This was used as an intraoperative method to check whether the constricting arch is effectively removed. Mediastinal pleura was left open unlike in other thoracic vascular surgeries, which prevented fibrosis and possible reconstriction on trachea.

In the post-operative period, the patient was extubated after 2.5 days of ventilatory support. Gavage feeding was started from the third and oral feeds from the sixth post-operative day. He was kept in the intensive care unit for 10 days and in the pediatric ward for 1 week during which he became completely free of stridor. At the 6-month follow-up, he was completely symptom free.

## DISCUSSION

Double aortic arch, although rare, is an important cause of persistent respiratory symptoms in infants. It is due to failure of regression of the right aortic arch. The remnant of the right aortic arch becomes the right innominate artery and leaves a left arch in normal development, freeing the trachea and esophagus. Failure of this process of absorption produces a vascular ring that completely encircles and compresses the esophagus and trachea, leading to severe respiratory and feeding difficulties. Usually, double aortic arch occurs without associated cardiovascular anomalies. However, anomalies like ventricular septal defect, tetralogy of Fallot, truncus arteriosus, transposition of great arteries, pulmonary atresia and complex univentricular defects are seen, with a reported incidence of 17%.[1,2] Respiratory symptoms at birth or during infancy should raise the possibility of vascular ring compression. Patients with double aortic arch can present with respiratory, gastrointestinal and cardiac symptoms. In a case series of 81 patients, 91% had respiratory, 40% had gastrointestinal and 28% had cardiac symptoms.[2] The common symptoms at presentation were stridor (77%), followed by wheezing, coughing, chest retractions, recurrent respiratory tract infection and apnea. The classic history in a patient with double aortic arch is noisy breathing, noted by parents in the first few weeks of life. Neonates may also have episodes of acute apnea and cyanosis, which is termed as ALTE (Acute life-threatening event). Gastrointestinal symptoms include choking while feeding, emesis, feeding difficulty and failure to thrive.

Diagnosis of double aortic arch can be made by echocardiography, however, it can be easily missed especially in those where the suprasternal images are not adequate. This is the case with many of the infants with double aortic arch due to presence of a stridor and respiratory distress. As the patients typically present with respiratory symptoms, bronchoscopy is usually the initial investigation that is performed. Pulsatile compression of the posterior and lateral wall of the trachea can be seen in such patients. However, MDCT with 3D reconstruction is the best single imaging technique for the diagnosis and characterization of vascular rings. It provides complete information regarding arterial branching pattern, location and extent of airway and esophageal obstruction and completely delineates cardiac anatomy. Successful imaging assists the cardiovascular surgeon in planning surgical management.

In conclusion, compression of the trachea and esophagus by vascular structures in childhood is uncommon and may be masked by non-specific respiratory symptoms. Thus, its diagnosis requires a high index of suspicion. MDCT should be performed on clinical suspicion in patients showing findings on diagnostic bronchoscopy or echocardiography. The introduction of ultrafast MDCT scanners with superior temporal and spatial resolution has facilitated surgical management of such patients by providing excellent visualization of cardiovascular structures with reconstructed 3D images.
